# Surgery, Anesthesia and Intensive Care Environment Induce Delirium-Like Behaviors and Impairment of Synaptic Function-Related Gene Expression in Aged Mice

**DOI:** 10.3389/fnagi.2020.542421

**Published:** 2020-09-25

**Authors:** Meghana Illendula, Hari Prasad Osuru, Bianca Ferrarese, Navya Atluri, Elzbieta Dulko, Zhiyi Zuo, Nadia Lunardi

**Affiliations:** ^1^Department of Anesthesiology, University of Virginia Health System, Charlottesville, VA, United States; ^2^Department of Anesthesiology and Intensive Care Medicine, University of Padova, Padua, Italy

**Keywords:** delirium, Intensive Care Unit, attention, memory, disorganized thinking, synuclein alpha, syntaxin 1a, neurotrophic receptor tyrosine kinase 1

## Abstract

**Objective:**

To establish a clinically relevant mouse model of perioperative delirium.

**Methods:**

Aged C57BL/6J mice were tested at baseline in the Y-maze novel arm preference, buried food, simple discrimination task of the attentional set-shifting test, and open field tests. They were subsequently randomized to insult (anesthesia, surgery, and Intensive Care Unit environment) or control group. Insult-exposed mice received laparotomy under sevoflurane anesthesia, propofol sedation and exposure to intermittent lights, sounds and cage shaking. Controls did not receive anesthesia, surgery, or intensive care environment. All mice were tested in the Y-maze novel arm preference, buried food, attentional, and open field tests at the end of intensive care environment (0 h) and every 6 h up to 24 h. Mouse hippocampi were collected at 24 h for gene expression analyses.

**Results:**

Surgery, anesthesia and Intensive Care environment decreased the entries in the Y-maze novel arm at 0 h (*P* = 0.001), 6 h (*P* < 0.001), 18 h (*P* = 0.002), and 24 h (*P* = 0.029). Insult exposure increased the latency to find a buried cereal reward at 18 h (*P* = 0.035) and 24 h (*P* = 0.027), and increased the trials to criterion in the reverse compound discrimination (*P* = 0.013) and extradimensional shift (*P* < 0.001) tasks of the attentional test. The overall incidence of delirium was 72% in A/S/I mice. Messenger RNA levels of synuclein alpha (−3.785 fold change relative to controls), Neurotrophic Receptor Tyrosine Kinase1 (−2.267), and syntaxin1a (−1.498) were decreased in the hippocampus of mice 24 h after insult exposure. Protein levels of syntaxin 1a (*P* = 0.012), Neurotrophic Receptor Tyrosine Kinase1 (*P* = 0.039), synuclein alpha (*P* = 0.017), phosphorylated synuclein alpha (*P* = 0.008), synaptophysin (*P* = 0.002), postsynaptic density protein 95 (*P* = 0.003), and microtubule-associated protein 2 (*P* = 0.013) were also decreased, relative to controls.

**Conclusion:**

Surgery, anesthesia and Intensive Care environment impaired mouse behaviors that depend on attention, memory, and thought organization. The changes were acute in onset and fluctuating in time. Mice with delirium exhibited decreased expression of key synaptic function-related genes. The behavioral changes induced by anesthesia, surgery, and Intensive Care environment in aged mice are consistent with the clinical features of human delirium, and support the use of this animal model for future mechanistic studies of perioperative delirium.

## Introduction

Perioperative delirium (POD) is an acute form of brain failure characterized by fluctuating consciousness, inattention, memory deficits, and disorganized thinking (Inouye, 2006; Deiner and Silverstain, 2009; Ansaloni et al., 2010; Jankowski et al., 2011; Hughes et al., 2012; Wang et al., 2016, 2018; Pandharipande et al., 2017; Evered et al., 2018). It occurs commonly in elderly patients in the Intensive Care Unit (ICU) after major surgery (Inouye, 2006; Ansaloni et al., 2010; Hughes et al., 2012; Wang et al., 2016, 2018; Pandharipande et al., 2017; Evered et al., 2018) and is associated with high in-hospital morbidity and mortality, greater institutionalization rates, and a steep trajectory of subsequent cognitive decline (Inouye, 2006; Deiner and Silverstain, 2009; Ansaloni et al., 2010; Jankowski et al., 2011; Hughes et al., 2012; Ren et al., 2015; Wang et al., 2016, 2018; Pandharipande et al., 2017). In the absence of a clear understanding of the biological processes underlying POD, targeted therapeutic options are scarce and largely ineffective (Deiner and Silverstain, 2009; Ansaloni et al., 2010; Hughes et al., 2012; Pandharipande et al., 2017).

A major barrier to advancing the understanding of the basic mechanisms of POD is the lack of adequate animal models. In fact, to date only few animal models for delirium research exist. Peng et al. (2016) assessed hippocampus-mediated attention, thought organization, and consciousness in young adult mice after a simple laparotomy. Culley et al. (2014) found impaired cognitive flexibility in the attentional set-shifting test in aged mice after lipopolysaccharide injection. Murray et al. (2012) used the paddling T-maze alternation task to capture memory deficits in mice with prion disease subjected to lipopolysaccharide injection. Anticholinergic drugs have also been employed to reproduce animal behavioral changes compatible with human delirium (Leavitt et al., 1994; Tamura et al., 2006; Field et al., 2012; Volgin et al., 2019). However, the translational significance of these animal models has been challenged by difficulties related to the use of young animals, the adoption of a short, simple laparotomy or exogenous lipopolysaccharide to produce inflammation, the use of a single behavioral test with long intervals between assessments, and a focus on a single putative pathway of delirium (i.e., acetylcholine deficiency).

Thus, there is a need for biologically and clinically relevant animal models that reproduce the realism of the clinical arena and faithfully recapitulate the multitude of intra- and post-operative risk factors for POD, including anesthesia, exacerbating surgical procedure, pain, the use of sedatives and sleep disruption in the ICU. As such, our goal was to establish a translationally relevant mouse model that could be used for studies on the basic mechanisms of POD. We subjected old mice to anesthesia, a complex laparotomy, and ICU environment (A/S/I) and deployed a battery of behavioral tests to evaluate for memory impairment, inattention, disorganized thinking, and cognitive inflexibility. Serial testing with short intervals between behavioral assessments enabled us to capture the acute onset and fluctuating course of POD. The hippocampi of A/S/I mice were collected following the last behavioral assessment and processed for gene expression studies. Our hypothesis was that A/S/I would induce changes in mouse behaviors consistent with the features of human POD and alter the expression of genes that are important for normal synaptic function.

## Materials and Methods

### POD Model Development

**FIGURE 1:**
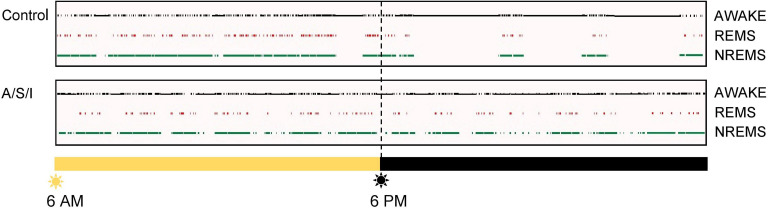
Representative hypnograms. Top panel: Rodents are nocturnal animals, i.e., they are asleep when lights are on, and are awake and active when lights are off. In line with this, the control mouse spent more time asleep, i.e., in NREMS and REMS, when lights were on, and more time awake after lights turned off. Bottom panel: The insult-exposed mouse continued to spend a substantial amount of time asleep after lights turned off, i.e., at a time when it should have been awake and active. REMS, rapid eye movement sleep; NREMS, non-rapid eye movement sleep.

All studies were approved by the Institutional Animal Care and Use Committee at the University of Virginia (Charlottesville, Virginia) and conducted in accordance with the National Institutes of Health (Bethesda, MD, United States). Sixty 18- to 20-month-old C57BL/6J male mice (Jackson laboratories, United States) were used. Mice from 8 litters were employed to control for litter variability. All experiments were started between 7 and 9 AM. Mice were randomly allocated to insult group or control group the morning of the experiment. Mice in the insult group (A/S/I) were subjected to 3 h of sevoflurane anesthesia during which a complex laparotomy was performed, followed by 2 h of sedation with propofol, and 12 h of ICU conditions. Mice in the control group were housed separately and did not receive A/S/I. In the insult group, anesthesia was titrated to a surgical plane with 3.0–3.5% sevoflurane in 100% oxygen via a nose cone. Under sterile conditions, a midline incision was made from the xiphoid to the symphysis, and skin, muscles and peritoneum were carefully dissected. After entering the abdominal cavity, the right and left descending colon, transverse colon, liver, and spleen were gently rubbed with a flexible ear loop for 5 min. The abdominal contents were left exposed to air for an additional 10 min, then incision was sutured layer-by-layer with 5-0 vicryl filaments. Laparotomy duration was 45–60 min, at what time the animal was moved into a chamber with sevoflurane 1.5–2.5% in 100% oxygen for up to 3 h, titrated to a toe pinch score of 1 (Lunardi et al., 2019). After 3 h, A/S/I mice received propofol 50–100 mg/kg intraperitoneally (i.p.). If needed, repeated doses of propofol (50 mg/kg i.p.) were administered. On average, mice received 100 mg/kg of i.p. propofol. Propofol was titrated to a righting reflex score of 1 and toe pinch scores above 1 (Lunardi et al., 2019). Gas concentrations were monitored during anesthesia and sedation with an inline Datex-Ohmeda S/5 gas analyzer (Datex Ohmeda, United States). Rectal temperature was maintained at 37 ± 0.5°C via a heating pad (PhysioSuite monitor, Kent Scientific, United States). Heart rate (HR), oxygen peripheral saturation (SpO_2_), and respiratory rate (RR) were monitored during anesthesia and sedation using a PhysioSuite monitor (Kent Scientific, United States). Mean HR, SpO_2_, and RR were 356 ± 63 beats/min, 95 ± 1%, and 92 ± 15 breaths/min, respectively. All physiologic measurements were performed by the same investigator. Analgesia was provided with subcutaneous bupivacaine (0.25%, 0.3–0.5 ml). Following propofol sedation, A/S/I mice were subjected to 12 h of ICU environment, consisting of exposure to a 33 W light, 90 dB sounds, and cage rattling via a horizontal shaker (120 rpm) for 1 min every 20 min. ICU environment was started between 12 PM and 2 PM, and terminated between 12 AM and 2 AM. Cage rattling was observed to consistently evoke arousal in quiescent A/S/I mice. To verify that cage rattling evoked sleep disruption, one control and one A/S/I mouse were surgically implanted with electroencephalographic and electromyographic electrodes as previously described (Lunardi et al., 2019) 3 weeks prior to A/S/I. Twenty-four hour recordings of sleep-wake states were obtained starting at the end of ICU environment. The hypnograms from these animals are shown in Figure 1.

### Neurocognitive Assessment

**FIGURE 2:**
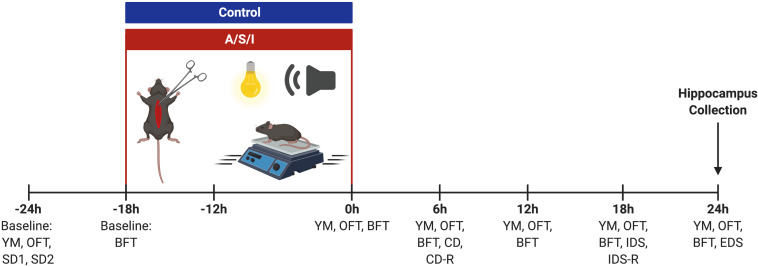
Schematic representation of the experimental design. YM, Y-maze novel arm preference test; OFT, open field test; SD, simple discrimination task; BFT, buried food test; CD, compound discrimination task; CD-R, reverse compound discrimination task; IDS, intra-dimensional shift task; IDS-R, reverse intra-dimensional shift; EDS, extra-dimensional shift task. Created with BioRender.com.

Mice were tested at the end of ICU environment (0 h) and every 6 h for up to 24 h by the same experimenter (see Figure 2 for a schematic of the experimental design). Mice were habituated to the behavioral testing room for at least 30 min prior to behavioral assessments. The behavioral apparatus was wiped clean with 70% alcohol between animals. The same cohort of mice was used for Y-maze novel arm preference test and open field tests. Two separate groups of animals were employed for the buried food and the attentional set-shifting tests, respectively. Data were recorded and analyzed with Any-Maze 5.0 software (Stoelting Co., United States).

#### Y-Maze Novel Arm Preference Test

The Y-maze novel arm preference protocol was adapted from previous studies (Peng et al., 2016; Wolf et al., 2016; Ji et al., 2017). The maze consisted of three arms (35 × 35 × 35 cm) with 120 degree angles between arms. Arms included the start arm, in which the mouse started to explore (always open); the novel arm, blocked during the first trial; and the third arm (always open). In the first trial, the mouse was placed in the start arm and explored the maze for 5 min with the novel arm closed. After 60 min, the mouse was placed back in the start arm and explored the maze for additional 5 min with the novel arm open. The number of entries into the novel arm during the second trial was used as a measure of spatial memory and attention.

#### Buried Food Test

The BFT was carried out as in previous investigations with modifications (Nathan et al., 2004; Lehmkuhl et al., 2014; Canugovi et al., 2015; Peng et al., 2016). Three days prior to A/S/I, mice were introduced to a sweet cereal. All food was removed from the cage 24 h prior to A/S/I and a BFT baseline was performed immediately preceding A/S/I. During the test, each mouse was placed in the center of a 25 × 18 × 13 cm cage filled with 3 cm of bedding, where a cereal pellet had been buried (0.5 cm below the bedding surface). The latency to eat the cereal (i.e., time from placement in the cage to the mouse grasping the pellet between teeth) was used as a measure of attention and organized thinking. A latency of 300 s was recorded when the mouse could not find the pellet within 5 min.

#### Attentional Set-Shifting Task

The AST was performed as in previous studies with modifications (Birrell and Brown, 2000; Culley et al., 2014; Heisler et al., 2015). Mice were food restricted to 85% of body weight and trained to dig for a cereal reward located in two ramekins placed inside the AST chamber for 2–3 weeks. The AST chamber consisted of a clear 20 × 30 × 20 cm box with a removable divider separating the starting point from the ramekins. Each ramekin was defined by two discrimination clues: the digging medium and a shape placed on the wall adjacent to the ramekin. A series of mediums and shapes (exemplars) was used throughout the test. Two standard discrimination (SD) tests were performed at baseline (24 h prior to A/S/I). In SD1 the relevant dimension was the medium, in SD2 it was the shape. Six hours after the end of ICU environment (6 h), mice were tested on the compound discrimination (CD) and the reverse CD (CD-R) tasks. CD used the same relevant medium exemplars as SD1, but combined it with a new irrelevant shape. In CD-R, the correct medium exemplar was switched. At 18 h mice were tested on the intra-dimensional shift (IDS) and reverse IDS (IDS-R) task. In the IDS task, a new medium was introduced as the relevant dimension, alongside a new irrelevant shape. In the IDS-R task, the correct medium exemplar was switched. At 24 h mice were tested on the extra-dimensional shift (EDS) task, where the relevant dimension was switched to shape, and a new medium and shape were introduced. The number of trials required to reach performance criteria (i.e., six consecutive correct choices, defined as the mouse vigorously digging in the pot with the cereal) was used to evaluate attention and cognitive flexibility. The order of relevant dimensions for each discrimination task and the assignment of specific exemplars for each task were the same between mice, in order to minimize inter-subject variability. Each trial lasted 5 min. AST time points (6, 18, and 24 h post ICU environment) were chosen based on the timing of the behavioral changes observed in the YM and BFT. Exemplars, relevant dimensions and time points for each AST task are presented in Table 1.

**TABLE 1:** Discrimination clues and timing used for the AST.

Time (hours post ICU)	Test	S^+^	S^–^	Relevant dimension
−24	SD1	Paper	Aspen	Medium
	SD2	Heart	Square	Shape
6	CD	Paper/Flower	Aspen/Triangle	Medium
	CD-R	Aspen/Flower	Paper/Triangle	Medium
18	IDS	Straw/Diamond	Cardboard/Cross	Medium
	IDS-R	Cardboard/Diamond	Straw/Cross	Medium
24	EDS	Wax paper/star	Cotton/Circle	Shape

#### Open Field Test

The open field test (OFT) was adapted from previous studies (Seibenhener and Wooten, 2015; Peng et al., 2016). Mice were allowed to explore a 40 × 40 × 40 cm box (MazeEngineers, United States) for 5 min. The total distance traveled by each mouse was used to evaluate mice’ motor performance.

### Gene Expression Studies

#### Tissue Collection

Following the last behavioral assessment (24 h), mice were subjected to brief sevoflurane anesthesia and their hippocampi were dissected on ice, snap-frozen in liquid nitrogen and stored at −80°C. We focused on the hippocampus because of its critical role in memory, thought processing and executive function (Frodl et al., 2006; Bird and Burgess, 2008; Susanne Shultz, 2010).

#### Screening of Gene Targets With Quantitative Polymerase Chain Reaction Arrays

Quantitative real-time polymerase chain reaction (qPCR) was performed to examine the expression level of genes associated with delirium, dementia, amnestic and cognitive disorders, using predesigned 96-well qPCR arrays (Tier 1–3 M96 panel, Bio-Rad, United States). Hippocampal total RNA was extracted from control (pooled *N* = 3) and A/S/I animals (pooled *N* = 3). cDNA synthesis was performed as previously described (Atluri et al., 2019). cDNA template (100 ng/well) was mixed with SsoAdvanced SYBR green 2× master mix and loaded (20 μl/per well) on predesigned 96-well qPCR array plates. PCR amplification was carried out on CFX Connect Real-Time PCR system as described by the manufacturer (Bio-Rad, United States). Data were normalized against the house keeping genes of the qPCR array plate. mRNA fold changes were expressed relative to control using Bio-Rad CFX manager analysis software. Selected differentially expressed genes [syntaxin1a (STX1a), neurotropomyosin receptor kinase 1 (Ntrk1), and synuclein alpha (Snca α)] were subsequently validated by immunoblotting.

#### Western Blotting

Western blot analysis was performed as described previously (Atluri et al., 2019). Briefly, hippocampal tissue from control (*N* = 5–7) and A/S/I animals (*N* = 5-7) was lysed in 0.2 ml ice-cold RIPA buffer in the presence of protease and phosphatase inhibitor cocktails (Thermo Scientific, United States). Protein concentration in the tissue lysate was measured using Pierce Protein Assay Kit (Thermo Scientific, United States). Tissue lysates (5–20 μg/lane) were separated by SDS-PAGE on 4–20% TGX-gradient gels (Bio-Rad, United States) and transferred onto nitrocellulose membranes (Millipore). Membranes were incubated for 1 h in SuperBlock (TBS) Blocking Buffer (Thermo Scientific, United States) at room temperature, followed by overnight incubation at 4°C with primary antibodies (Cell-signaling): Snca α (D37A6) Rabbit mAb # 4179 (1:3000); phospho-Snca α (Ser129, D1R1R) Rabbit mAb # 23706 (1:3000); STX1a (D4E2W) Rabbit mAb # 18572 (1:3000); Ntrk1 Antibody # 2505 (1:1000); SYP Antibody Rabbit pAb #4329 (1:1000); PSD-95 Rabbit mAb #3450 (1:1000); MAP2 Rabbit pAb #4542 (1:1000) and beta-tubulin Mouse mAb # 66240 (Proteintech, United States). Membranes were washed with TBS-Tween and incubated with horseradish peroxidase-conjugated secondary antibodies: anti-rabbit IgG or anti-mouse IgG (1:10,000, cell signaling) for 1 h at room temperature. Membranes were washed, immunoreactivity was detected using chemiluminescence substrate (SuperSignal West Femto, Thermo Scientific, United States), and scanned using a G-box (Syngene, United States). Protein intensity was measured densitometrically using Gene Tools software (Syngene, United States). Changes in protein expression level were normalized to beta-tubulin and expressed as percent change to controls.

### Immunofluorescence

Immunofluorescent staining was performed on paraffin embedded tissue sections. Briefly, paraffin embedded hippocampal sections were de-paraffinized in xylene and rehydrated through a graded series of ethanol-water mixtures. Heat-induced epitope retrieval (HIER) was performed using 10 mM sodium Citrate (pH 6.0) and 0.05% Tween-20 solution for 20 min at 95°C. After HIER, tissue sections were permeabilized with 0.3% triton X-100 and blocked with 5% normal goat serum and 1% bovine serum albumin (BSA) in TBST (Tris-buffered saline containing 0.1% Triton X-100) for 1 h at room temperature, then incubated overnight at 4°C with PSD-95 monoclonal antibody (Cat. #3450, Cell signaling) diluted 1:300 in TBS and 1% BSA. Tissue sections were washed in TBS buffer containing 0.02% Tween-20, then incubated with donkey anti-rabbit IgG secondary antibody conjugated to Alexa Fluor 488 (#A21206, Invitrogen) for 1 h at room temperature in dark light. Sections were washed in TBS buffer and mounted with fluoromount-G anti-fade mounting medium (Southern Biotechnology Associates, Inc.). Sections were examined using a ZEISS AxioImager.Z1 (Carl ZEISS Microimaging, NY).

### Statistical Analysis

Two animals died during surgery and therefore their data were not used for analysis. One insult-exposed animal was in pain and therefore not tested at the 12 h time point for the YM novel arm preference test and OFT. Prism 8 (GraphPad software, Inc., La Jolla, CA, United States) was used for all statistical analyses. *P* < 0.05 was considered statistically significant.

#### Behavioral Studies

Based on prior investigations that found impairments in AST, BFT, and YM performance in mice with POD (Culley et al., 2014; Peng et al., 2016), we estimated that A/S/I and control group would need 10 mice each to detect a minimum difference in means of 30% with an expected standard deviation of residuals at 20% and a desired power of 0.8. We used the Shapiro–Wilk test to assess normality and found that our behavioral data were not normally distributed. We used two-tailed Mann–Whitney *U* test to compare A/S/I and control groups in the YM, BFT, and OFT. Two way repeated measures ANOVA was used for the AST test. Data are expressed as median percentage of baseline behavior ± S.E.M. for the YM, BFT, and OFT. Data are expressed as mean number of trials to performance criteria ± S.E.M. for the AST.

#### *Z* Score

We used the same method as in human studies to calculate the *Z* score for the diagnosis of delirium (Monk et al., 2008; Zhong et al., 2020). Briefly, in order to obtain a *Z* score for each individual animal at each time point of behavioral assessment, we subtracted the postoperative test results from baseline (pre-A/S/I) data and divided the result by the corresponding S.D. generated from the control group. The sign was adjusted so that negative *Z* scores indicated deterioration from baseline test. For the AST test, we considered the SD1 and SD2 tests as baseline, and treated the CD, CD-R, IDS, IDS-R, and EDS as separate tests. The OFT was not included in the calculation of the *Z* score, as there were no differences in OFT performance between A/S/I and control mice. One *Z* score equal to or less than −1.96 at any time point of behavioral assessment indicated a mouse displaying delirium-like behaviors. For the calculation of the overall incidence of delirium in our animal model, a mouse was classified as exhibiting delirium if at least two *Z* scores across the 24 h testing period were equal to or less than −1.96.

#### Molecular Studies

Power calculations were based on two-tailed *t*-test of mean difference using preliminary data that quantified Snca α from A/S/I and control mice. Based on those data, it was estimated that a group size of *N* = 14 (7 mice per group) would provide 90% power to detect a 30% reduction in Snca α levels. Western blot data were compared with two-tailed unpaired *t*-test. Data are presented as mean ± S.D.

## Results

### Effects of Anesthesia/Surgery/ICU on Mouse Behavior in the Y-Maze Novel Arm Preference Test

**FIGURE 3:**
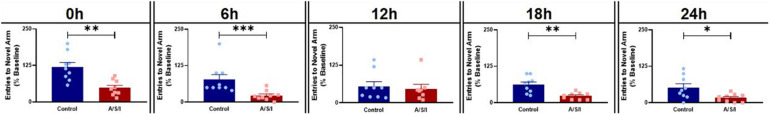
Effects of Anesthesia/Surgery/ICU on mice behavior in the Y-maze novel arm preference test. A/S/I decreased the number of entries in the novel arm of the Y-maze relative to controls at the end of ICU environment (0 h, *P* = 0.0012), and at 6 h (*P* = 0.001), 18 h (*P* = 0.002) and 24 h (*P* = 0.029) post ICU environment. There were no differences in the number of entries in the Y-maze novel arm between A/S/I and control mice at 12 h post ICU environment (*P* = 0.588). Two-tailed Mann–Whitney *U* test (*N* = 9 A/S/I and 9 control mice for all time point, except *N* = 8 A/S/I and *N* = 9 control mice for the 12 h time point). ^∗^*P* < 0.05, ^∗∗^*P* < 0.01, and ^∗∗∗^*P* < 0.001.

We first assessed the effects of A/S/I on spatial memory and attention by employing the YM novel arm preference test. As shown in Figure 3, A/S/I significantly decreased the number of entries in the YM novel arm compared to the control group at 0 h: 45.0% ± 8.75 in A/S/I versus 114.3% ± 15.63 in control (*U* = 6, *P* = 0.001), 6 h: 20.0% ± 5.65 in A/S/I vs 50.0% ± 16.98 in control (*U* = 5.5, *P* = 0.001), 18 h: 25.0% ± 3.92 in A/S/I versus 67.0% ± 9.27 in controls (*U* = 8, *P* = 0.002) and 24 h: 20.0% ± 4.63 in A/S/I versus 42.9% ± 13.03 in controls (*U* = 16, *P* = 0.030). The number of novel arm visits was unchanged in A/S/I mice relative to controls at 12 h: 41.50% ± 14.65 in A/S/I versus 50.0% ± 15.28 in controls (*U* = 30, *P* = 0.590). These data suggest that A/S/I mice experienced acute and fluctuating impairments in spatial memory and attention.

### Effects of Anesthesia/Surgery/ICU on Mouse Behavior in the Buried Food Test

**FIGURE 4:**
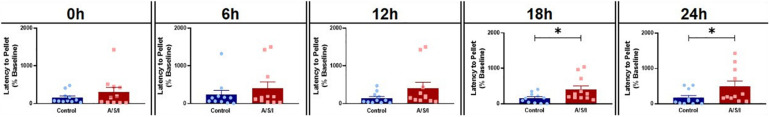
Effects of Anesthesia/Surgery/ICU on mice behavior in the buried food test. A/S/I mice took significantly longer to find a buried sweet cereal compared to control mice at 18 h (*P* = 0.035) and 24 h (*P* = 0.027) post ICU environment. There was no difference in the latency to find the buried cereal between A/S/I and control mice at 0 h (*P* = 0.950), 6 h (*P* = 0.480) and 12 h (*P* = 0.116) after the end of ICU environment. Two-tailed Mann-Whitney *U* test (*N* = 11 A/S/I and 11 control mice). ^∗^*P* < 0.05.

We next assessed the effects of A/S/I on attention and thought organization by means of the BFT. As shown is Figure 4, A/S/I mice took significantly longer to find the buried cereal pellet compared to controls at 18 h: 280.4% ± 101.7 in A/S/I versus 158.1% ± 42.01 in control (*U* = 28.5, *P* = 0.035) and 24 h: 210.0% ± 147.9 in A/S/I versus 84.9% ± 63.97 in control (*U* = 27, *P* = 0.027). There were no differences in latency to find the food at 0 h: 161.1% ± 126.2 in A/S/I versus 80.95% ± 46.04 in control (*U* = 59, *P* = 0.950), 6 h: 155.6% ± 167.6 in A/S/I versus 152.2% ± 113.8 in control (*U* = 49, *P* = 0.480), and 12 h: 166.7% ± 161.0 in A/S/I versus 86.96% ± 45.37 in control (*U* = 36, *P* = 0.116). These findings indicate that A/S/I impaired mouse’s ability to find a buried cereal reward, a behavior that relies on intact attention and orderly thinking.

### Effects of Anesthesia/Surgery/ICU on Mouse Behavior in the Attentional Set-Shifting Task

**FIGURE 5:**
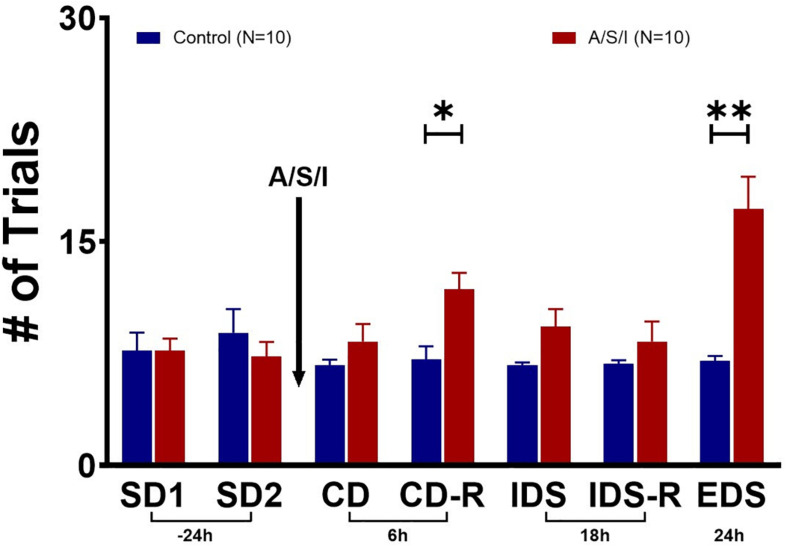
Effects of Anesthesia/Surgery/ICU on mice behavior in the attentional set-shifting test. There were no differences in baseline performance in the SD tasks between A/S/I and control mice (SD1: *P* > 0.999; SD2: *P* = 0.973). While A/S/I and control mice performance on the CD task was not significantly different (*P* = 0.834), insult-exposed mice required a significantly higher number of trials to achieve performance criteria in the CD-R task (*P* = 0.028) compared to controls. There were no differences in performance in the IDS and IDS-R tasks between A/S/I and control mice (*P* = 0.337 and *P* = 0.921, respectively). A/S/I mice experienced difficulties finding the reward in the EDS task compared to controls (*P* = 0.007). SD, simple discrimination; CD, compound discrimination; CD-R, reverse compound discrimination; IDS, intra-dimensional shift; IDS-R, reverse intra-dimensional shift; EDS, extra-dimensional shift. Two-way repeated measures ANOVA (*N* = 10 A/S/I and 10 control mice). ^∗^*P* < 0.05 and ^∗∗^*P* < 0.01.

Since A/S/I impaired hippocampus-mediated behaviors that depend on memory, attention and organized thinking, we asked whether prefrontal cortex-mediated cognition could also be compromised in A/S/I mice. Repeated measures ANOVA demonstrated a main effect of A/S/I treatment [*F* (1, 18) = 10.46, *P* = 0.005], a main effect of test time [*F* (2.595, 46.71) = 5.427, *P* = 0.004], and an interaction of these factors [*F* (6, 108) = 7.206, *P* < 0.0001]. Sidak’s multiple comparisons test showed significant effects of A/S/I on the CD-R (*P* = 0.028) and the EDS (*P* = 0.007) tasks. As shown in Figure 5, in the CD-R task, A/S/I mice took 11.80 ± 1.10 trials to find the reward cereal versus 7.10 ± 2.80 trials in controls. In the EDS task, it took A/S/I mice 17.20 ± 2.15 trials versus 7.00 ± 0.33 trials in controls. A/S/I did not affect performance in the SD, CD, IDS or IDS-R tasks (SD1: *P* > 0.999; SD2: *P* = 0.973; CD: *P* = 0.834; IDS: *P* = 0.337; IDS-R: *P* = 0.921). These data indicate that A/S/I mice experienced difficulties with tasks that required a reversal of the dimension associated with the reward or a shift in attention toward a new dimension, which is consistent with a phenotype of impaired attention and cognitive flexibility.

### Effects of Anesthesia/Surgery/ICU on Mouse Motor Performance in the Open Field Test

**FIGURE 6:**
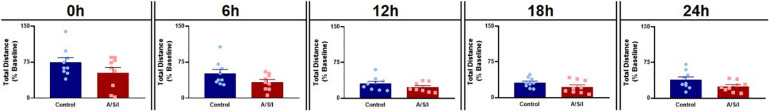
Effects of Anesthesia/Surgery/ICU on mice motor performance in the Open Field test. There were no differences in total distance traveled between A/S/I and control mice at any of the time points studied post ICU environment (0 h: *P* = 0.490; 6 h: *P* = 0.168; 12 h: *P* = 0.246; 18 h: *P* = 0.142; 24 h: *P* = 0.081). Two-tailed Mann–Whitney *U* test (*N* = 9 A/S/I and 9 control mice for all time points, except *N* = 8 A/S/I and *N* = 9 control mice for the 12 h time point).

To rule out the possibility that motor disability may have been responsible for the changes in behavior we observed, we assessed the effects of A/S/I on mouse motor performance in the OFT. As shown in Figure 6, we found no differences in total distance traveled between A/S/I and control mice (0 h: 63.0% ± 11.10 versus 63.0% ± 9.94, *U* = 32, *P* = 0.490; 6 h: 33.0% ± 5.51 versus 38.06% ± 8.54, *U* = 24.5, *P* = 0.169; 12 h: 18.50% ± 3.63 versus 30.0% ± 4.68, *U* = 23.5, *P* = 0.246; 18 h: 19.6% ± 4.42 versus 31.0% ± 3.48, *U* = 23.5, *P* = 0.142; 24 h: 23.0% ± 3.04 versus 31.0% ± 6.30, *U* = 20.5, *P* = 0.081, in A/S/I and control mice, respectively). These findings suggest that the behavioral abnormalities we observed were not due to impairment of mice motor performance.

### Incidence of Delirium After Anesthesia/Surgery/ICU

To determine the incidence of delirium in our animal model, we calculated a *Z* score for the diagnosis of delirium for each individual mouse (Monk et al., 2008; Zhong et al., 2020). This approach identified individual mice with delirium by comparing the changes in the test scores of an individual mouse after A/S/I with changes in the test scores of the control group over the same time interval. As shown in Table 2, at 0 h, 1/17 (5.9%) mice in the A/S/I group and 0/17 (0%) mice in the control group displayed delirium-like behaviors (i.e., had one *Z* score less than or equal to −1.96). At 6 h, 8/25 (32.0%) A/S/I mice and 2/25 (8.0%) control mice exhibited delirium-like behaviors. At 12 h, the number of A/S/I mice with delirium-like behaviors was 9/17 (52.9%), compared to 2/17 (11.7%) in the control group. At 18 and 24 h, the number of A/S/I mice with delirium-like behavior was 13/25 (52.0%) and 20/25 (80.0%), while that of control mice with delirium was 7/25 (28.0%) and 3/25 (12.0%), respectively. These data indicate that more mice in the A/S/I group developed cognitive decline than control mice, regardless of what time point of behavioral assessment was considered. Specifically, the overall incidence of delirium was 72%, as a total of 18 out of 25 A/S/I mice exhibited a *Z* score less than or equal to −1.96 in at least two behavioral assessments across the 24 h testing period [versus a total of 3/25 (12%) in the control group].

**TABLE 2:**
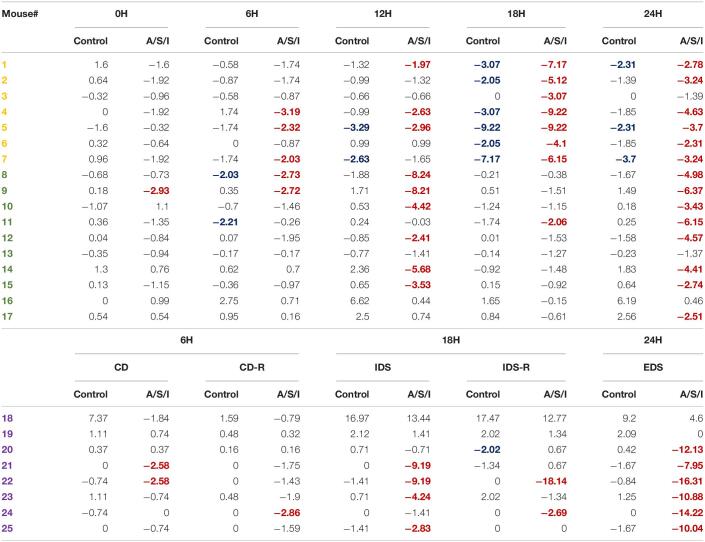
Overall incidence of delirium after Anesthesia/Surgery/ICU. Only A/S/I and control mice that performed behavioral assessments in a paired format were included in *Z* score calculations. The number of each individual mouse was randomly assigned. Negative *Z* scores refer to cognitive impairment. Numbers in red indicate A/S/I mice with delirium-like behaviors as identified by one *Z* score less than or equal to −1.96. Numbers in blue indicate control mice with delirium-like behaviors as identified by one *Z* score less than or equal to −1.96 (*N* = 17–24 A/S/I and 17–24 controls). Mice in yellow were tested on the YM novel arm preference test. Mice in green were tested on the BFT. Mice in purple were tested on the AST.

### Effects of Anesthesia/Surgery/ICU on the Gene Expression and Protein Level of Synuclein Alpha, Syntaxin 1a, and Neurotrophic Receptor Tyrosine Kinase 1 Genes

**FIGURE 7:**
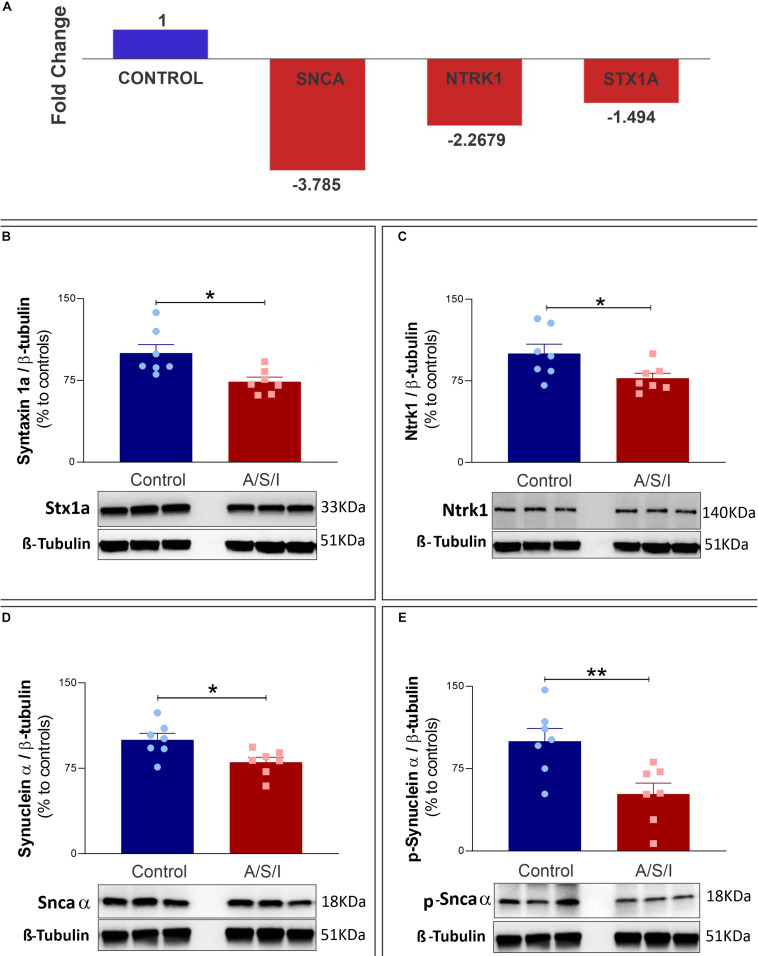
Effects of Anesthesia/Surgery/ICU on expression of synuclein alpha, neurotrophic receptor tyrosine kinase 1 and syntaxin 1a genes. **(A)** Snca α, Ntrk1, and STX1a mRNA levels were significantly impaired in A/S/I mice relative to controls following ICU environment (–3.785, –1.498, and –2.268 fold decrease, respectively). Hippocampal total RNA was extracted from A/S/I animals (pooled *N* = 3) and controls (pooled *N* = 3). **(B–E)** Protein levels of STX1a (**B:** *P* < 0.012), Ntrk1 (**C:** *P* = 0.039), and Snca α (**D:** *P* = 0.017) were significantly decreased in A/S/I mice relative to controls. Snca phosphorylated at serine residue 129 was also significantly down-regulated in insult-exposed mice (**E:** *P* = 0.008). Two-tailed unpaired *t*-test (*N* = 7 A/S/I and *N* = 7 control mice). STX1a, syntaxin 1a; NtrK1, neurotrophic tropomyosin receptor kinase 1; Snca α, synuclein alpha; p-Snca α, synuclein alpha phosphorylated at serine residue 129. ^∗^*P* < 0.05 and ^∗∗^*P* < 0.01, respectively.

The synuclein alpha (Snca α), syntaxin 1a (STX1a), and neurotrophic receptor tyrosine kinase 1 (Ntrk1) genes code for proteins that are critical in the organization of pre-synaptic vesicles, release of neurotransmitters, and synaptic plasticity (Cabin et al., 2002; Aboulkassim et al., 2011; David and Subhojit, 2012; Watanabe et al., 2013; Ortega et al., 2018; Josephy-Hernandez et al., 2019). Since A/S/I-induced inattention, memory impairment and disorganized thinking may be due to synaptic dysfunction, we examined the effects of A/S/I on hippocampal mRNA expression of Snca α, STX1a and Ntrk1. We found that Snca α, STX1a and Ntrk1 mRNA levels were significantly reduced in A/S/I mice relative to controls (−3.785, −1.498, and −2.268 fold decrease, respectively; Figure 7A). Since A/S/I impaired hippocampal transcription of Snca α, STX1a, and Ntrk1 genes, we asked whether their protein levels would also be compromised in A/S/I mice. Immunoblotting of Snca α, STX1a, and Ntrk1 revealed a decrease in the band densities of A/S/I mice compared to controls. There was no significant difference in β-tubulin band densities in the hippocampus of A/S/I and control mice. Quantification of the western blots, based on the ratio of STX1a, Ntrk1, and Snca α levels to β-tubulin, showed that STX1a, Ntrk1, and Snca α protein levels were significantly decreased in A/S/I mice relative to controls (Figures 7B–D: STX1a: *P* = 0.013; Ntrk1: *P* = 0.039; Snca α: *P* = 0.018). Since increased levels of phosphorylated Snca α have been associated with postoperative delirium in humans (Sunwoo et al., 2013), we also tested whether protein levels of Snca α phosphorylated at serine residue 129 [p-Snca α, a post-translationally modified form of Snca α commonly found in synucleinopathy lesions (Fujiwara et al., 2002; Kim et al., 2018)], was altered in A/S/I mice relative to controls. We found that p-Snca α was significantly reduced in insult-exposed mice (51% in A/S/I versus 100% in control mice, *P* = 0.008; Figure 7E). These findings suggest that A/S/I induced hippocampal impairment of STX1a, Ntrk1, Snca α, and p-Snca α at both transcriptional and translational levels.

### Effects of Anesthesia/Surgery/ICU on Synaptophysin, Microtubule-Associated Protein 2 and Postsynaptic Density Protein 95 Levels

**FIGURE 8:**
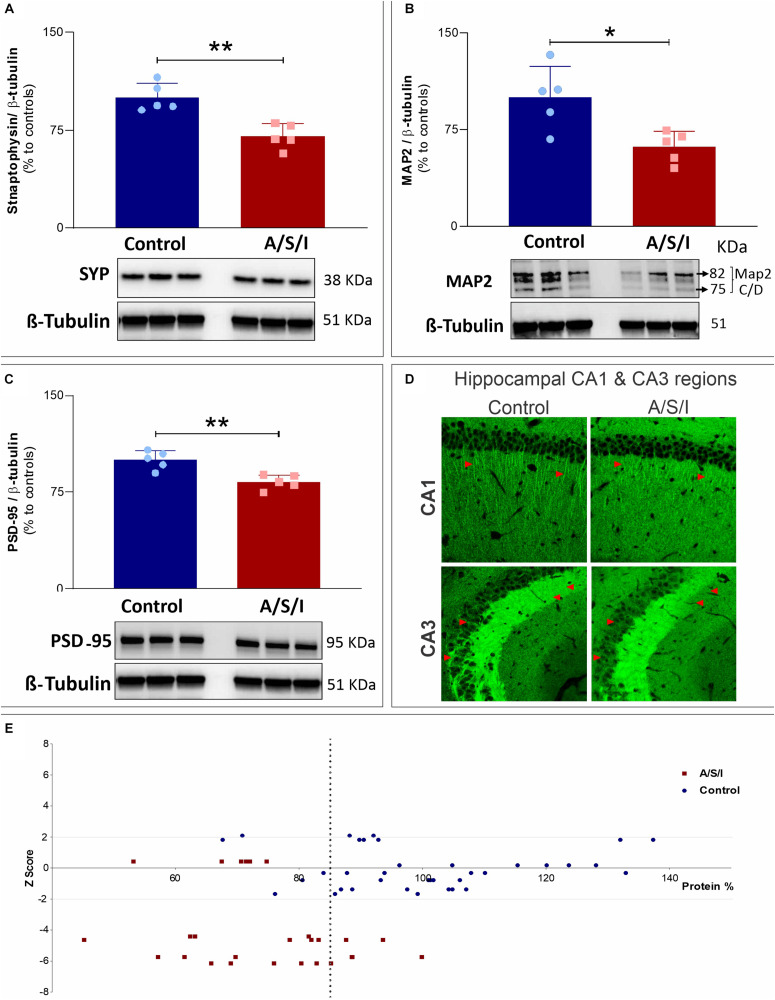
Effects of Anesthesia/Surgery/ICU on synaptophysin, microtubule-associated protein 2 and postsynaptic density protein 95 levels. **(A–C)** Protein levels of Syp (**A:** *P* = 0.002), MAP2 (**B:** *P* = 0.013), and PSD-95 (**C:** *P* = 0.003) were significantly decreased in A/S/I mice relative to controls. Two-tailed unpaired *t*-test (*N* = 5 A/S/I and *N* = 5 control mice). Syp, synaptophysin; MAP2, microtubule-associated protein 2; PSD-95, postsynaptic density protein 95. **(D)** Representative 20× magnification images of the distribution of PSD-95 immunostaining in sagittal CA1 and CA3 hippocampal sections from two control and two A/S/I mice. Arrows highlight differences in PSD-95 immunofluorescent staining. **(E)** Plot of STX1a, Ntrk1, Snca α, Syp, MAP2, and PSD-95 protein levels against individual mouse *Z* scores. *Z* scores from behavioral assessments obtained at the 24 h time point were used, since tissue collection for protein studies was performed immediately after this behavioral assessment. The vertical dotted line represents a protein level of 85% (expressed as change from control). ^∗^*P* < 0.05 and ^∗∗^*P* < 0.01, respectively.

Since our hypothesis was that A/S/I would induce changes in the expression of key synaptic function-related proteins, we examined the protein levels of three additional proteins that are pivotal for synaptic function, i.e., synaptophysin (SYP), microtubule-associated protein 2 (MAP2), and postsynaptic density protein 95 (PSD-95). These proteins are involved in a number of critical roles for synaptic formation, maintenance and plasticity, including vesicle exocytosis, facilitation of membrane receptor anchorage, and endocytosis of synaptic vesicles (Teng et al., 2001; Kwon and Chapman, 2011; Vallejo et al., 2017). Immunoblotting of SYP, MAP2, and PSD-95 revealed a decrease in the band densities of A/S/I mice compared to controls. Of note, MAP2 immunoblotting revealed two isoforms (isoform C and D) with close molecular weight (82 and 75 KDa, respectively). These isoforms were quantified as one densitometric band. There was no significant difference in β-tubulin band densities in the hippocampus of A/S/I and control mice. Quantification of the western blots, based on the ratio of SYP, MAP2 and PSD-95 levels to β-tubulin levels, showed that the levels of all three proteins were significantly decreased in A/S/I mice relative to controls (Figures 8A–C; SYP: *P* = 0.002; MAP2: *P* = 0.013; PSD-95: *P* = 0.003). In order to assess for a possible separation in the distribution of synaptic protein values vis-à-vis behavioral impairments in control and A/S/I mice, we plotted all protein values from all measured synaptic markers against individual *Z* scores at the 24 h behavioral assessment time point (i.e., the time of hippocampi collection for protein quantification studies) from control mice and A/S/I mice with delirium. We found that the distribution of synaptic marker protein levels in relation to individual *Z* scores was very different in control and A/S/I mice. As shown in Figure 8, panel E, the range of protein content for STX1a, Ntrk1, Snca α, SYP, MAP2, and PSD-95 was between 67.61 and 137.36% in control mice, and only 5 out of 37 synaptic marker levels were below 85% in control mice. No control mice with these levels of synaptic markers exhibited delirium-like behaviors (i.e., had at least one *Z* score equal to or less than −1.96). Meanwhile, the range of synaptic marker content was much lower in A/S/I mice, between 45.15 and 99.88%, and 21 out of 26 synaptic marker values were below 85% in A/S/I mice. Importantly, of the 21 synaptic markers below 85% in A/S/I mice, 15 (71.43%) were associated with *Z* scores indicative of profound behavioral impairment, i.e., four standard deviations or more below baseline.

## Discussion

The goal of this study was to establish a translationally relevant mouse model of POD that could be used for further investigations of its basic mechanisms. Our hypothesis was that a combination of anesthesia, complex laparotomy, and ICU environment would induce changes in mouse behaviors consistent with the clinical features of human POD. Since inattention, memory impairment, and disorganized thinking may be due to synaptic dysfunction, we also hypothesized that delirium-like behaviors would be associated with impairment in the expression of key genes required for normal synaptic activity. We found that A/S/I altered mouse behaviors that depend on the integrity of attention, memory, and thought organization, three important features of human POD. The delirium-like behavioral changes were acute in onset and fluctuating in time, and therefore consistent with the time course of clinical POD. We also found that STX1a, Ntrk1, Snca α, SYP, MAP2 and PSD-95 – key proteins for the regulation of synaptic vesicle trafficking, neurotransmitter release and synaptic plasticity – were significantly down-regulated in A/S/I mice compared to controls.

The mouse model we developed improves significantly over previous animal models of POD (Leavitt et al., 1994; Tamura et al., 2006; Field et al., 2012; Murray et al., 2012; Culley et al., 2014; Ren et al., 2015; Peng et al., 2016; Volgin et al., 2019). First, it employs senescent mice equivalent to human subjects 60 to 70 years old (Dutta and Sengupta, 2016; The Jackson Laboratory,, 2017), i.e., ages associated with greater risk of POD (Inouye, 2006; Deiner and Silverstain, 2009; Ansaloni et al., 2010; Wang et al., 2016). Second, the methods used to test the animals are consistent with the ICU-Confusion Assessment Method (ICU-CAM), a widely used ICU algorithm (Ely et al., 2001; Pandharipande et al., 2007; Fagundes et al., 2012; Gusmao-Flores et al., 2012; Peng et al., 2016) that evaluates key aspects of delirium, i.e., acute onset and fluctuating course, inattention, and disorganized thinking. Third, compared to previous studies (Murray et al., 2012; Culley et al., 2014; Ren et al., 2015; Peng et al., 2016), our model uses multiple behavioral tests and shorter intervals between assessments to capture the acute and fluctuating nature of POD. It also combines several behavioral tests to assess hippocampus- and prefrontal cortex-mediated changes in the cognitive domains of attention, thought process, and cognitive flexibility. Last, our model faithfully recapitulates the perioperative setting and accounts for the multifactorial pathogenesis of POD by combining several known intra- and post-operative risk factors for delirium (Inouye, 2006; Deiner and Silverstain, 2009; Ansaloni et al., 2010; Hughes et al., 2012; Wang et al., 2016), i.e., anesthesia, major surgery, pain, use of sedatives, and sleep disruption in the ICU.

First, we found that A/S/I decreased the number of entries into the novel arm of the YM. The YM novel arm preference test is a widely accepted test to assess hippocampus-dependent spatial memory (Sarnyai et al., 2000; Akwa et al., 2001; Yau et al., 2007; Sanderson and Bannerman, 2010; Kraeuter et al., 2019). Mice with spatial memory deficits tend to enter the novel arm less often than normal mice, as a result of impaired discrimination between previously seen and novel objects (Akwa et al., 2001; Sanderson and Bannerman, 2010; Kraeuter et al., 2019). Of relevance, spatial memory requires the presence of attention and organized thinking (Sanderson and Bannerman, 2010; Peng et al., 2016; Kraeuter et al., 2019). Therefore, our finding that A/S/I decreased the number of entries into the novel arm of the YM suggests that A/S/I mice developed memory deficits, inattention, and confused thinking associated with POD. Next, A/S/I mice took significantly longer to find a buried cereal reward in the BFT. The BFT assesses mice’ olfactory memory by exploiting their reliance upon olfactory clues to forage for food (Lehmkuhl et al., 2014; Peng et al., 2016; Zheng et al., 2019). By testing for mice’s ability to find a hidden cereal pellet, it also evaluates for the presence of intact attention and orderly thinking (Peng et al., 2016; Zheng et al., 2019). Hence, our BFT findings support the idea that A/S/I mice developed impairments in the domains of attention and thought process.

It is postulated that the inattention observed in patients with POD results from defective cross-talk between prefrontal and parietal cortices (Corbetta and Shulman, 2002; Choi et al., 2012; Culley et al., 2014). As such, behavioral tests mediated by the prefrontal cortex are well suited to study delirium in animals (Culley et al., 2014; Carr et al., 2019). The AST is commonly used in patients to detect attention and executive function impairments secondary to prefrontal cortex dysfunction (Culley et al., 2014), and the circuits underlying behavior during the AST are highly conserved across rodents and humans (Heisler et al., 2015). In our model, A/S/I mice performed below the level of controls in those AST tasks that required the ability to modify a response when the rules had changed, i.e., switch to a reward-associated dimension that was previously irrelevant or form an attentional set with a new pair of discrimination clues. In those tasks, A/S/I mice exhibited a continued choice using the previously learned rule, consistent with a phenotype of cognitive inflexibility, inattention, and impaired executive function.

The overall incidence of delirium in our animal model, identified by a *Z* score equal to or less than −1.96 in at least two behavioral assessments, was 72%. This rate is similar to that reported in elderly patients admitted to the ICU after a major surgery (Inouye, 2006; Ansaloni et al., 2010; Hughes et al., 2012; Vasilevskis et al., 2012; Wang et al., 2016, 2018; Pandharipande et al., 2017; Evered et al., 2018). Importantly, no A/S/I mouse exhibited a *Z* score less than −1.96 at each and every behavioral assessment. Rather, the most common scenario among A/S/I mice was that of a *Z* score equal to or less than −1.96 in 2 out of 5 behavioral sessions. This suggests that A/S/I-induced changes in spatial memory, attention, and thought process fluctuated in severity over time, in agreement with the ICU-CAM concept. Notably, 12% of control mice experienced delirium. This percentage is in line with that reported for hospitalized general medical patients (11–25%) (Vasilevskis et al., 2012) who do not undergo anesthesia or surgery, and are not admitted to the ICU. We ascribe these behavioral changes to stress/sleep disruption due to frequent handling during repeated behavioral assessments.

Syntaxin1a is a pre-synaptic protein with a central role in vesicle docking/fusion and neurotransmitter release (Watanabe et al., 2013; Craig et al., 2015; Troup et al., 2019). It is also involved in short-term synaptic plasticity, which in turn is essential for stabilization of neural circuit function (Ortega et al., 2018). Similarly, Ntrk1 activity is important for synaptic plasticity, neuronal survival, and proliferation (Bruno et al., 2004; Saragovi, 2005; Parikh et al., 2013), and Ntrk1 receptors are implicated in spatial learning and memory (Aboulkassim et al., 2011; Josephy-Hernandez et al., 2019). Snca α’s primary physiologic role is thought to be in regulating vesicle release and synaptic plasticity (Cabin et al., 2002; Scott and Roy, 2012; Vargas et al., 2014). Recent studies have also indicated that Snca α may play a role in neuroprotection from oxidative stress, DNA repair, and ATP synthesis (Musgrove et al., 2013; Ludtmann et al., 2016; Schaser et al., 2019). Similarly, SYP, MAP2 and PSD-95 are major elements of synaptic dendritic spines and play a central role in synapse development, stability and plasticity (Teng et al., 2001; Vallejo et al., 2017). In the setting of defective levels of STX1a, Ntrk1, Snca α, SYP, MAP2 and PSD-95, it is a plausible hypothesis that disruption of neurotransmitter release and impaired synaptic plasticity, alongside compromised neuronal survival and increased susceptibility to perioperative stressors, may alter neural circuit function and lead to the loss of memory and attention, disorganized thinking, and cognitive inflexibility observed in A/S/I mice. Importantly, we found that the distribution of synaptic marker protein levels in relation to individual *Z* scores was very different in control and A/S/I mice, with control mice exhibiting on average higher protein levels and no delirium-like behaviors versus A/S/I mice displaying lower levels of synaptic marker proteins associated with profoundly significant behavioral impairments. This differential distribution of synaptic protein values vis-à-vis behavioral impairments in control and A/S/I mice supports the notion that decreased levels of STX1a, Ntrk1, Snca α, SYP, MAP2, and PSD-95 may be relevant to the behavioral impairments observed in mice with POD. Further studies are needed to corroborate the hypothesis that changes in these proteins are the underlying mechanism of POD.

Interestingly, Ren et al. (2015) found increased Snca α levels in the cortex of young female mice 12 h after a simple laparotomy and anesthesia. However, surgery plus anesthesia in our study did not increase Snca α levels 24 h after surgery. The reason behind the discrepancy between these data and our results is not known at the present time. It is possible it may be due to differences in animal age and gender, experimental protocol, brain area, and timing of Snca α measurement.

Our study has limitations. First, since our experimental protocol consisted of a combination of insults, we cannot draw conclusions on the relative contribution of each insult to the behavioral abnormalities we observed. However, our primary objective was to develop an animal model that recapitulates the complexity of the perioperative environment as it relates to delirium pathogenesis, rather than to focus on the effect of each insult on delirium phenotype. Further studies are needed to dissect the impact of each component of our protocol. Second, we employed male mice in our experiments. This was done to conceptually establish the model and since previous studies found that male gender is a predisposing factor for human delirium (Inouye, 2006). In future studies, we will use the established system to test the effects of female sex on A/S/I-induced behavior and biochemical changes. Third, we only assessed the effects of A/S/I on synaptic function-related proteins in the hippocampus. A/S/I may have different effects on these proteins in other regions of the brain. Future studies should assess the impact of A/S/I on Snca α, SYT1a, Ntrk1, SYP, MAP2, and PSD-95 in other brain regions, using our established system.

In conclusion, our data collectively show that in aged mice, a combination of anesthesia, complex surgery, and ICU environment impaired memory, attention, thought organization, and cognitive flexibility with acute onset and fluctuating course. Delirium-like behaviors were also associated with impairment in the expression of genes that are required for synaptic stability, plasticity and overall function. Given that sudden, waxing-and-waning inattention and disorganized thinking are core features of POD, A/S/I-induced behavioral changes are consistent with clinical human delirium and support the use of our model for mechanistic studies of POD.

## Data Availability Statement

The datasets generated for this study are available on request to the corresponding author.

## Ethics Statement

The animal study was reviewed and approved by Animal Care and Use Committee University of Virginia.

## Author Contributions

NL and ZZ conceived the project. MI, BF, and NL designed the behavioral studies. HO and NL designed the molecular studies. MI, BF, HO, NA, ED, and NL performed the experiments. MI, HO, and NL wrote the manuscript. All authors reviewed the manuscript.

## Conflict of Interest

The authors declare that the research was conducted in the absence of any commercial or financial relationships that could be construed as a potential conflict of interest.
